# Antiplatelet agents maintain arteriovenous fistula and graft function in patients receiving hemodialysis: A nationwide case–control study

**DOI:** 10.1371/journal.pone.0206011

**Published:** 2018-10-18

**Authors:** Yung-Ho Hsu, Yu-Chun Yen, Yi-Chun Lin, Li-Chin Sung

**Affiliations:** 1 Division of Nephrology, Department of Internal Medicine, Shuang Ho Hospital, Taipei Medical University, New Taipei City, Taiwan; 2 Department of Internal Medicine, School of Medicine, College of Medicine, Taipei Medical University, Taipei, Taiwan; 3 Research center of Biostatistics, College of Management, Taipei Medical University, Taipei, Taiwan; 4 Division of Cardiology, Department of Internal Medicine, Shuang Ho Hospital, Taipei Medical University, New Taipei City, Taiwan; International University of Health and Welfare, School of Medicine, JAPAN

## Abstract

**Background:**

In this study, we evaluated the effects of various medications on the patency of vascular access (VA) for hemodialysis.

**Methods:**

We analyzed data from the Longitudinal Health Insurance Database of Taiwan. We adopted a case–control study design within a cohort of patients who had received regular hemodialysis between 2002 and 2012; 34,354 patients with first VA failure were identified, and the duration from VA creation date to the first VA failure date was calculated. We then classified these patients into two groups, namely arteriovenous fistula (AVF, *n* = 25,933) and arteriovenous graft (AVG, *n* = 8,421). Each group was further divided into two subgroups, namely short-term (<1 year) and long-term (≥1 year) patency.

**Results:**

The risk factors for early VA failure were age ≥65 years, diabetes mellitus, hyperlipidemia, cerebral vascular disease, congestive heart failure, peripheral artery disease, and sepsis. Male sex, hypertension, cancer, and peptic ulcer were associated with early AVF failure. Antiplatelet therapy increased the AVF and AVG patency times with adjusted odds ratios of 0.748 (95% confidence interval [CI]: 0.703–0.796, *p* < 0.0001) and 0.810 (95% CI: 0.728–0.901, *p* = 0.0001), respectively. A significant decrease in the VA failure risk was observed with an increase in the cumulative defined daily dose of antiplatelet agents.

**Conclusion:**

This nationwide study demonstrated that some risk factors were associated with early VA failure and that the use of antiplatelet agents prevented the loss of VA patency in a dose–response manner. Thus, antiplatelet drugs should be routinely administered to high-risk patients receiving dialysis.

## Introduction

Functional vascular access (VA) is essential for hemodialysis (HD), and VA can be achieved using an arteriovenous fistula (AVF), an arteriovenous graft (AVG), or a central venous catheter. VA failure is a major cause of morbidity among patients receiving HD, and it accounts for the hospitalization of approximately 20% of these patients [1–4]. An AVF is the preferred type of VA for HD because the complication rates and patient survival rates associated with AVFs are lower and higher, respectively, than those of associated with AVGs or central venous catheters [5, 6]. The 1-year patency rates of native AVFs are extremely variable, ranging from 40% to 80% [7]. The most crucial cause of AVF failure is progressive neointimal hyperplasia at the venous anastomosis, which results in stenosis and subsequent thrombosis [8–10]. An AVG is generally cannulated more easily and can be used for HD sooner after surgery than an AVF. However, in AVGs, stenosis frequently develops at the graft–vein anastomosis, thus leading to access thrombosis [11]. Central venous catheters are used for rapid access in immediate dialysis and are associated with relatively high rates of infection and complications.

The mechanisms of VA failure are not completely understood; however, studies have shown that inflammation may play a critical role [7, 12–14]. Several clinical studies have reported that antiplatelet drugs improve VA patency rates [10, 11, 15, 16]. A recent Cochrane review reported that antiplatelet treatment can improve the 1-month patency rates of AVFs and AVGs [10]. Two meta-analyses have revealed that aspirin prevents thrombosis of AVFs but not AVGs [17, 18]. Some in vitro and in vivo studies have demonstrated that statins reduce neointimal proliferation, vascular inflammation, and VA failure [4, 16, 19, 20]. However, one retrospective analysis that used data from the United States Renal Data System (USRDS) reported that statins are not associated with significant reduction in VA failure risk and that antiplatelet agents are associated with a significantly increased risk of AVF dysfunction [21]. These inconsistent findings might have resulted partially from differences in methodology, clinical factors, and populations among the studies. Percutaneous transluminal angioplasty (PTA) and surgical thrombectomy are the two principal treatments for patients with progressive AVF or AVG failure [7, 8], which may affect VA survival time. Some trials have not considered the effects of PTA or surgical history on VA survival time.

The long-term efficacy of drug therapy in preventing the first AVF or AVG dysfunction in Taiwanese patients with chronic HD has not been reported thus far. We conducted a nationwide population-based case–control study by using the claims data of patients between 2001 and 2012 from the National Health Insurance Research Database (NHIRD) of Taiwan. We investigated the associations between various drug regimens and patency rates after VA creation.

## Methods

The National Health Insurance (NHI) program has been operational in Taiwan since 1995 and provides comprehensive health insurance coverage for all residents of Taiwan. Currently, the NHI program includes 97% of all hospitals and clinics in Taiwan. In addition, 98% of the >23 million potential enrollees are covered under the NHI program [22]. We studied the entire population of Taiwan between 2001 and 2012, and the follow-up period extended to the end of 2012.

### Database

In this study, we obtained medical insurance claims data from the Health and Welfare Data Science Center (HWDC), Ministry of Health and Welfare of Taiwan. These data constitute the medical information of each insured individual in Taiwan. Data files from the NHI claims database contain registration numbers and original claims data for reimbursement. All the data are anonymous and are deidentified by scrambling the identification codes of both the patients and medical facilities, thus rendering the NHI reimbursement data suitable for academic research [23]. The International Classification of Disease, Ninth Revision, Clinical Modification (ICD-9-CM) codes are used for reporting diagnoses. Thus, the diagnoses in the NHI claims database of Taiwan exhibit high accuracy and validity. Taiwanese studies have demonstrated the high accuracy and validity of ICD-9-CM diagnoses in the Taiwan’s NHI claims database [22, 23]. This study was approved by the Joint Institutional Review Board of Taipei Medical University (TMU-JIRB N201609023; see S1 File) and supervised by the HWDC.

### Study population

**Fig 1:**
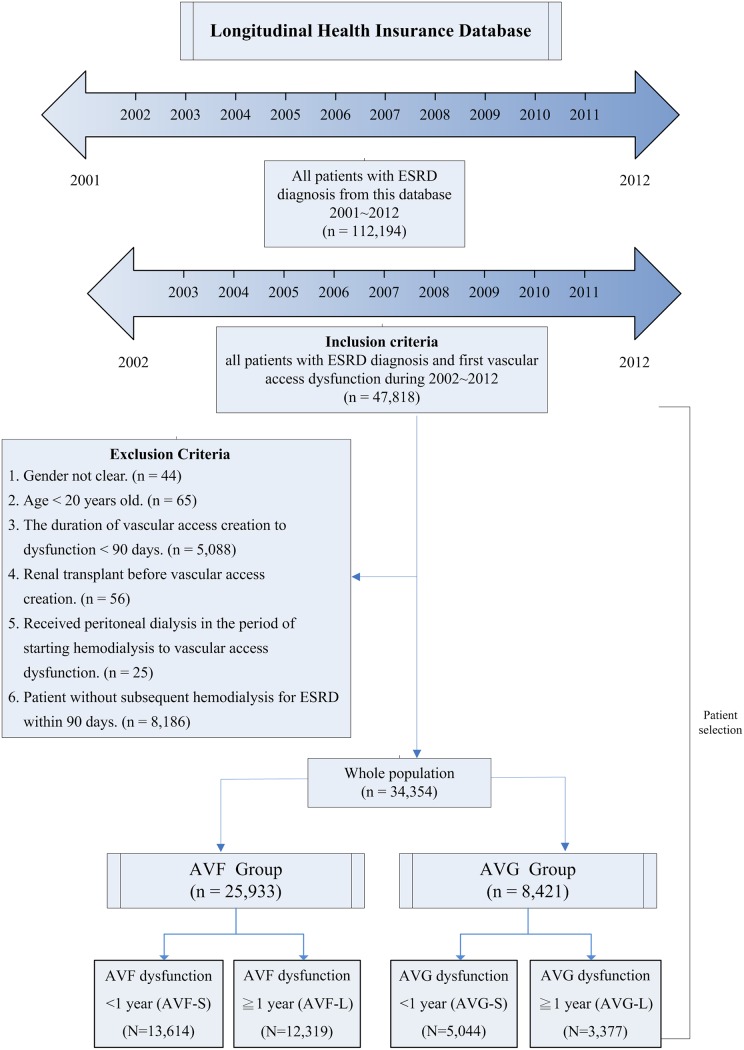
Flowchart of selection criteria and process of patients receiving hemodialysis and experienced vascular access (VA) dysfunction. The time of occurrence of VA dysfunction was used to define two subgroups, namely short-term (S) (<1 year) and long-term (L) (≥1 year) VA dysfunction. AVF, arteriovenous fistula; AVG, arteriovenous graft; ESRD, end-stage renal disease.

This study used a case–control design. A flowchart of the patient selection procedure is presented in Fig 1. From the Registry for Catastrophic Illness Patients Database, a subdatabase of the NHI claims database, we identified all patients with end-stage renal disease (ESRD) (ICD-9-CM code: 585) who had received HD between January 1, 2001, and December 31, 2012 (*n* = 112,194). We included all patients with ESRD and first VA dysfunction between 2002 and 2012 (*n* = 47,818). VA dysfunction was defined as VA failure that occurs >3 months after the initial HD; this criterion excluded patients with very early VA failure (*n* = 5,088). The causes of very early VA failure differ from those associated with failure >3 months; therefore, patients with very early failure were excluded [24]. We retrospectively searched for the first VA-related procedure (surgical thrombectomy or PTA) codes, which could be considered surrogates for first VA failure because stable patients on HD would receive these procedures only after VA failure. Patients who received renal transplantation (*n* = 56) or peritoneal dialysis (*n* = 25), either before or after HD, were excluded. In addition, we excluded patients if they had unclear sex data (*n* = 44), were younger than 20 years (*n* = 65), or had received HD for <90 days (*n* = 8,186). The final study cohort comprised 34,354 patients. We divided the patients into two groups, namely AVF and AVG. Each group was further divided into two subgroups according to the duration between VA creation date and first VA failure date, namely short-term (S) (<1 year) and long-term (L) (≥1 year) patency.

### Definition of comorbidities

The comorbidities of the patients included in this study were hypertension (HTN), diabetes mellitus (DM), coronary artery disease (CAD), congestive heart failure (CHF), chronic obstructive pulmonary disease (COPD), cerebral vascular disease (CVD), hyperlipidemia, peripheral artery disease (PAD), peptic ulcer disease (PUD), sepsis or systemic inflammatory response syndrome (SIRS), and cancer (Tables 1 and 2). The comorbidities were identified using ICD-9-CM codes [25]. The existing comorbidities of the patients were defined using the following criteria: ICD-9-CM codes appearing in the inpatient database at least once within 1 year before initial HD, diagnoses of the comorbidity in the outpatient database at least twice, and timestamps of first and last diagnoses at least 30 days apart within 1 year before HD initiation.

**Table 1:** Baseline characteristics of AVF patients with VA dysfunction.

	VA dysfunction within 1 year (AVF-S) (*N* = 13,614)		VA dysfunction after 1 year (AVF-L) (*N* = 12,319)		*p* value ^a^
	*n*	(%)	*n*	(%)	
Sex					0.0311*
Male	7340	(53.92)	6477	(52.58)	
Female	6274	(46.08)	5842	(47.42)	
Age (years)					<0.0001*
20–40	625	(4.59)	778	(6.32)	
41–50	1554	(11.41)	1754	(14.24)	
51–60	3144	(23.09)	3172	(25.75)	
61–70	3860	(28.35)	3288	(26.69)	
71–80	3439	(25.26)	2618	(21.25)	
>80	992	(7.29)	709	(5.76)	
Comorbidities					
DM	8639	(63.46)	6816	(55.33)	<0.0001*
HTN	12436	(91.35)	10993	(89.24)	<0.0001*
Hyperlipidemia	4150	(30.48)	3495	(28.37)	0.0002*
CAD	4915	(36.10)	4069	(33.03)	<0.0001*
CVD	2423	(17.80)	1892	(15.36)	<0.0001*
CHF	4392	(32.26)	3442	(27.94)	<0.0001*
COPD	2184	(16.04)	1846	(14.98)	0.0189*
PAD	1061	(7.79)	547	(4.44)	<0.0001*
Cancer	1998	(14.68)	1678	(13.62)	0.0150*
PUD	4051	(29.76)	3347	(27.17)	<0.0001*
Sepsis/SIRS	2012	(14.78)	1363	(11.06)	<0.0001*
Medications					
Statins	2128	(15.63)	1741	(14.13)	0.0007*
Antiplatelets	5136	(37.73)	4723	(38.34)	0.3096
Anticoagulants	389	(2.86)	416	(3.38)	0.0160*
Dipyridamole	1454	(10.68)	1091	(8.86)	<0.0001*

AVF, arteriovenous fistula; CAD, coronary artery disease; CHF, congestive heart failure; COPD, chronic obstructive pulmonary disease; CVD, cerebral vascular disease; DM, diabetes mellitus; HTN, hypertension; L, long-term patency; PAD, peripheral artery disease; PUD, peptic ulcer disease; S, short-term patency; SIRS, systemic inflammatory response syndrome; VA, vascular access.

^a^ *p* value was estimated using chi-squared test.

* *p* < 0.05.

**Table 2:** Baseline characteristics of AVG patients with VA dysfunction.

	VA dysfunction within 1 year (AVG-S) (*N* = 5,044)		VA dysfunction after 1 year (AVG-L) (*N* = 3,377)		*p* value ^a^
	*n*	(%)	*n*	(%)	
Sex					0.5988
Male	1957	(38.80)	1291	(38.23)	
Female	3087	(61.20)	2086	(61.77)	
Age (years)					< .0001*
20–40	136	(2.70)	83	(2.46)	
41–50	346	(6.86)	278	(8.23)	
51–60	849	(16.83)	641	(18.98)	
61–70	1348	(26.72)	979	(28.99)	
71–80	1669	(33.09)	1059	(31.36)	
>80	696	(13.80)	337	(9.98)	
Comorbidities					
DM	3359	(66.59)	2096	(62.07)	<0.0001*
HTN	4665	(92.49)	3116	(92.27)	0.7153
Hyperlipidemia	486	(9.64)	297	(8.79)	0.1931
CAD	2077	(41.18)	1312	(38.85)	0.0329*
CVD	1179	(23.37)	686	(20.31)	0.0009*
CHF	1960	(38.86)	1106	(32.75)	<0.0001*
COPD	991	(19.65)	582	(17.23)	0.0054*
PAD	468	(9.28)	209	(6.19)	<0.0001*
Cancer	849	(16.83)	519	(15.37)	0.0744
PUD	1764	(34.97)	1079	(31.95)	0.0041*
Sepsis/SIRS	1081	(21.43)	578	(17.12)	<0.0001*
Medications					
Statins	780	(15.46)	523	(15.49)	0.9770
Antiplatelets	2161	(42.84)	1422	(42.11)	0.5040
Anticoagulants	204	(4.04)	135	(4.00)	0.9148
Dipyridamole	464	(9.20)	285	(8.44)	0.2301

AVG, arteriovenous graft; CAD, coronary artery disease; CHF, congestive heart failure; COPD, chronic obstructive pulmonary disease; CVD, cerebral vascular disease; DM, diabetes mellitus; HTN, hypertension; L, long-term patency; PAD, peripheral artery disease; PUD, peptic ulcer disease; S, short-term patency; SIRS, systemic inflammatory response syndrome; VA, vascular access.

^a^ *p* value was estimated using chi-squared test.

* *p* < 0.05.

### Medication use and risk of VA failure

Some medications, including statins, antiplatelet agents, anticoagulants, and dipyridamole, that have been reported to affect VA outcome were examined [4, 10, 11, 15, 16, 19, 20, 26–29]. All prescription information was extracted from the NHIRD. The prescription claims of candidate medications were tracked for up to 1 year before the first VA dysfunction date by using NHIRD drug codes (see S2 File). The defined daily dose (DDD) recommended by the World Health Organization is a unit for measuring a prescribed amount of drug [30]. It is the assumed average maintenance dose per day of a drug consumed for its main indication in adults. In this study, cumulative DDD (cDDD) was defined as a time-independent variable in which the daily supply of each prescription dispensed was summed over time within a 90-day period before the first VA failure date. To examine the drug–response relationship, we further categorized the medications of each candidate into two groups within the AVF and AVG groups (<10 and ≥10 cDDD in the anticoagulants for their rapid onset of action, or <30 and ≥30 cDDD in other drugs). The patients who used drugs for <30 or <10 cDDD were defined as drug nonusers (Table 3).

**Table 3:** Comparison of VA dysfunction risk at <1 year and >1 year after VA creation in AVF and AVG patients who used different medications.

	AVF Patients			AVG Patients		
	aOR ^a^	95% CI	*p* value	aOR ^a^	95% CI	*p* value
Medications ^b^						
Statins cDDD ≥ 30	0.977	(0.876–1.089)	0.6727	0.831	(0.687–1.004)	0.0548
Antiplatelets cDDD ≥ 30	0.748	(0.703–0.796)	<0.0001*	0.810	(0.728–0.901)	0.0001*
Anticoagulants cDDD ≥ 10	1.009	(0.770–1.321)	0.9502	1.446	(0.887–2.357)	0.1389
DipyridamolemcDDD ≥ 30	1.022	(0.742–1.408)	0.8948	0.787	(0.468–1.322)	0.3655
Characteristic						
Age ≥ 65 years	1.318	(1.252–1.388)	<0.0001*	1.195	(1.090–1.310)	0.0001*
Male	1.080	(1.027–1.136)	0.0026*	1.045	(0.954–1.145)	0.3459
Comorbidities						
DM	1.349	(1.278–1.423)	<0.0001*	1.215	(1.103–1.339)	<0.0001*
HTN	1.091	(1.001–1.189)	0.0467*	0.911	(0.768–1.081)	0.286
Hyperlipidemia	1.416	(1.271–1.578)	<0.0001*	1.266	(1.045–1.533)	0.0159*
CAD	1.023	(0.966–1.083)	0.4358	1.011	(0.917–1.115)	0.8264
CVD	1.078	(1.007–1.154)	0.0318*	1.143	(1.025–1.274)	0.0162*
CHF	1.145	(1.082–1.213)	<0.0001*	1.263	(1.145–1.394)	<0.0001*
COPD	0.985	(0.919–1.056)	0.6661	1.083	(0.965–1.216)	0.1767
PAD	1.730	(1.553–1.927)	<0.0001*	1.497	(1.260–1.777)	<0.0001*
Cancer	1.098	(1.023–1.180)	0.0102*	1.113	(0.985–1.257)	0.0852
PUD	1.076	(1.018–1.137)	0.0098*	1.090	(0.992–1.198)	0.0728
Sepsis/SIRS	1.326	(1.231–1.429)	<0.0001*	1.249	(1.115–1.400)	0.0001*

cDDD, cumulative defined daily dose; aOR, adjusted odds ratio; AVG, arteriovenous graft; AVF, arteriovenous fistula CAD, coronary artery disease; CHF, congestive heart failure; COPD, chronic obstructive pulmonary disease; CVD, cerebral vascular disease; DM, diabetes mellitus; HTN, hypertension; L, long-term patency; PAD, peripheral artery disease; PUD, peptic ulcer disease; S, short-term patency; SIRS, systemic inflammatory response syndrome; VA, vascular access.

^a^ AOR was estimated by multiple logistic regression with all variables listed in Table 2 in the model.

^b^ Specific medications with <10 or 30 cDDD as the drug nonuser group.

* *p* < 0.05.

### Statistical analysis

Baseline demographic and comorbidity characteristics are presented as proportions as appropriate and were compared using chi-squared statistics. We used a logistic model to estimate the odds ratios (ORs) and 95% confidence intervals (CIs) for the association between drug use and risk of VA dysfunction. The adjusted OR (aOR) was estimated using multiple logistic regression; the ORs were adjusted for sex, age group, comorbidities, and medication. We also performed a stratified analysis to identify the dose–response effect in both the VA groups (Tables 4 and 5). Furthermore, we divided the cDDDs of antiplatelet agents into four group variables, namely cDDD = 0, 1 < cDDD < 30, 31 < cDDD < 90, and cDDD > 90, to compare the VA failure risk in the different cDDD groups by using multiple logistic regression estimation. The data were analyzed using SAS statistical software (version 9.3 for Windows; SAS Institute Inc., Cary, NC, USA). The results were considered statistically significant if two-tailed *p* < 0.05.

**Table 4:** Comparison of VA dysfunction risk at <1 year and >1 year after VA creation in AVF patients who received different doses of antiplatelet drugs.

	AVF patients				
	Number of patients		Multiple logistic regression		
	VA dysfunction within 1 year (AVF-S)	VA dysfunction after 1 year (AVF-L)	Adj. OR ^a^	95% CI	*p* value
	*n* (%)	*n* (%)			
Medications					
Antiplatelet cDDD = 0 (ref.)	8478 (62.27)	7597 (61.67)	1.000	-	-
0 < Antiplatelet cDDD ≤ 30	2140 (15.72)	1793 (14.55)	0.985	(0.917–1.057)	0.6689
30 < Antiplatelet cDDD ≤ 90	1864 (13.69)	1827 (14.83)	0.756	(0.700–0.815)	<0.0001*
90 < Antiplatelet cDDD	1132 (8.31)	1102 (8.95)	0.728	(0.662–0.800)	<0.0001*

AVF, arteriovenous fistula; cDDD, cumulative defined daily dose; L, long-term patency; S, short-term patency; VA, vascular access.

^a^ Adj. OR (adjusted odds ratio) was estimated using multiple logistic regression and adjusted for all other variables in Table 2.

* *p* < 0.05.

**Table 5:** Comparison of VA dysfunction risk at <1 year and >1 year after VA creation in AVG patients who received different doses of antiplatelet drugs.

	AVG patients				
	Number of patients		Multiple logistic regression		
	VA dysfunction within 1 year (AVG-S)	VA dysfunction after 1 year (AVG-L)	Adj. OR ^a^	95% CI	*p* value
	*n* (%)	*n* (%)			
Medications					
Antiplatelet cDDD = 0 (ref.)	2883 (57.16)	1955 (57.89)	1.000	-	-
0 < Antiplatelet cDDD ≤ 30	882 (17.49)	502 (14.87)	1.111	(0.980–1.261)	0.1013
30 < Antiplatelet cDDD ≤ 90	790 (15.66)	561 (16.61)	0.862	(0.758–0.982)	0.0254*
90 < Antiplatelet cDDD	489 (9.69)	359 (10.63)	0.794	(0.678–0.929)	0.0042*

AVF, arteriovenous graft; cDDD, cumulative defined daily dose; L, long-term patency; S, short-term patency; VA, vascular access.

^a^ Adj. OR (adjusted odds ratio) was estimated using multiple logistic regression and adjusted for all other variables in Table 2.

* *p* < 0.05.

## Results

### Baseline characteristics of the AVF and AVG groups

The baseline characteristics of the AVF and AVG groups in the study cohort of 34,354 patients are listed in Tables 1 and 2. Among the 25,933 AVF patients, we identified 13,614 and 12,319 patients who did (AVF-S) and did not (AVF-L), respectively, experience AVF failure (and require intervention) within 1 year after the index date of VA creation. Among the 8,421 AVG patients, we identified 5,044 and 3,377 patients who did (AVG-S) and did not (AVG-L), respectively, experience AVG failure within 1 year after the index date of VA creation (Fig 1). In the patients with VA dysfunction, the proportion of men was higher among the patients with AVF than among those with AVG (53.3% vs 38.6%). The short-term patency groups, AVF-S and AVG-S, exhibited higher mean ages and significantly higher prevalence rates of comorbidities than the long-term patency groups, AVF-L and AVG-L. Furthermore, more patients in the AVF-S group used dipyridamole and statins than did in the AVF-L group. However, no differences in prescribed medications were observed between the AVG-S and AVG-L groups (Tables 1 and 2).

### Risk factors for early VA dysfunction

After adjustment for potential confounders through multiple logistic regression, early VA dysfunction remained associated with advanced age, DM, hyperlipidemia, CVD, CHF, PAD, sepsis or SIRS (all aOR > 1 and *p* < 0.05). In addition, male sex and the comorbidities of HTN, cancer, and PUD were associated with early AVF failure but not early AVG failure (Table 3).

### Outcomes in medication users and nonusers

Among the four types of medications, only antiplatelet agent users exhibited significantly longer patency durations than drug nonusers after adjustment for related covariates (Table 3). Antiplatelet agent use resulted in longer durations of AVF and AVG patency, with aORs of 0.748 (95% CI: 0.703–0.796, *p* < 0.0001) and 0.810 (95% CI: 0.728–0.901, *p* = 0.0001), respectively (Table 3).

An analysis of antiplatelet agent users based on cDDD within the 90-day period before the first VA failure date revealed a dose–response effect (Tables 4 and 5). When the AVF-group patients were stratified according to the cDDD of the medications, the aORs for AVF failure were 0.985 (95% CI: 0.917–1.057, *p* = 0.6689), 0.756 (95% CI: 0.700–0.815, *p* < 0.0001), and 0.728 (95% CI: 0.662–0.800, *p* < 0.0001) for the patients (drug users) who received cDDDs of 0–30, 30–90, and >90 compared with the drug nonusers. In this stratified analysis, prevention of early AVF failure was associated with a cDDD of antiplatelet agent use of ≥30 (Table 4). The antiplatelet agents conferred a similar protective effect in the AVG-group patients (Table 5).

## Discussion

The key new findings of this nationwide cohort study are as follows. (1) The number of patients with the first VA dysfunction and the male-to-female ratio were higher in the AVF group than in the AVG group. (2) The clinical predictors of the first VA dysfunction were advanced age, hyperlipidemia, DM, CVD, CHF, PAD, and sepsis or SIRS. (3) The additional risk factors for the first AVF failure were male sex, HTN, cancer, and PUD. (4) After clinical risk adjustment, the use of antiplatelet agents at cDDD ≥30 prevented loss of VA patency.

Maintaining VA patency remains one of the most crucial challenges in patients who receive HD. The pathogenesis of very early AVF failure (<3 months) remains poorly understood [14]. By excluding this group of patients, we ensured that the study did not include patients with immature fistulas; thus, we improved the reliability of our findings. Furthermore, the strength and advantage of our study is that we used data of almost the entire national population of Taiwan. Because our study involved a large number of participants who were followed-up for a long period, our results provide real-world evidence [31]. The Taiwan NHIRD dataset has been proven to be highly reliable; therefore, our results are highly convincing [31, 32].

The most common cause of VA dysfunction is venous stenosis, caused by venous neointimal hyperplasia within the juxta-anastomotic region (for an AVF) or at the graft–vein junction (for an AVG). The following factors contribute to neointimal hyperplasia: (1) surgical trauma at the time of VA creation, (2) hemodynamic shear stress at the artery–vein or graft–vein junctions, (3) bioincompatability of the AVG, (4) vessel injury caused by dialysis needle punctures, and (5) endothelial dysfunction caused by uremia toxin [14, 33]. A review article divided the risk factors for AVF dysfunction into two categories, namely nonmodifiable factors, such as advanced age, DM, hypotension, arteriosclerosis, and artery and venous anatomical factors, and modifiable factors, such as smoking, anastomosis type, cannulation technique, and first cannulation being performed within 14 days of VA creation [34]. Although numerous studies have examined the clinical, anatomical, and technical factors affecting VA dysfunction, the causes of individual variation in numerous patients remain unknown [34–38]. Additional studies on comprehensive risk identification and adjustment are essential for improving the accuracy of comparisons.

Reports have revealed that female sex is an independent risk factor for primary AVF failure, which is contrary to our results [37, 39, 40]. In addition, female sex was associated with AVF maturation failure in one European cohort study [41]. Women exhibit smaller vessel diameters and higher vasomotor activity than men; these findings can explain the poorer outcome of AVFs in women than in men [37, 39, 40]. However, some data have suggested that the 1-year patency rates in women are similar to those in men for AVFs [34, 42]. Our study revealed a higher proportion of AVG creation in women than in men (Table 1). The discrepancy between the results of the previous studies and those of the present study is possibly caused by the exclusion of patients with very early VA failure (<3 months) from our analysis as well as differences in selection criteria for VA creation and demographics.

The role of adjuvant medications in prolonging VA patency is a crucial research topic. Several clinical investigations and trials have reported that statins, anticoagulants, or antiplatelet drugs can reduce thrombosis and improve VA outcomes [9–11, 15, 16, 20, 43–45]. In this study, only the use of antiplatelet agents was associated with longer VA patency durations among patients receiving HD after adjustment for multiple clinical risk factors, thus supporting the evidence obtained from several randomized studies and one related study in the Cochrane database of systematic review [10, 11, 15, 45]. If the dose of the antiplatelet agent was >30 cDDD, the aOR for the ability of the antiplatelet agent to reduce VA dysfunction risk in patients receiving HD was significant. This is also the first study to propose that antiplatelet agents exert effects in a dose-responsive manner in patients receiving HD who are antiplatelet users.

Some neutral or contradictory results have been reported. Meta-analyses by Coleman et al. and Palmer et al. have shown that antiplatelet therapy prevented only AVF thrombosis and did not exert an effect on AVG thrombosis [17, 18]. However, the quality of the evidence was low because of the heterogeneity among the trials, small number of studies used in each comparison, short follow-up periods, and moderate methodological quality of the studies resulting from incomplete reporting. The USRDS Dialysis Mortality and Morbidity Wave II Study investigated 901 patients in a retrospective study and concluded that the risk of AVF failure associated with the use of antiplatelet agents was significantly higher than that associated with other medications [16, 21]. However, the doses and dates for initiation and withdrawal of the antiplatelet drugs were not recorded. Furthermore, patients who receive antiplatelet drugs usually exhibit more severe comorbidities than do patients who do not receive this treatment, which could have introduced bias. The clinical risk adjustment was limited to age, sex, race, CAD history, and PAD history.

Several possible factors may explain the beneficial effect of antiplatelet agents. The pathogenesis of venous neointimal hyperplasia has been elucidated previously; oxidative stress, platelet activation, endothelial dysfunction, and inflammation play a crucial role in the development of venous neointimal hyperplasia [13, 14, 21, 35, 38, 46]. Aspirin not only acts as an antiplatelet agent but also reduces oxidative stress and inflammation. Antiplatelet agents may be associated with superior cardiovascular outcomes than other medications in patients receiving HD, thus reducing intradialytic hypotension and the stasis and hypercoagulability of blood. Additional studies are warranted to verify the effects of antiplatelet agents on VA patency and to explore the underlying mechanism.

Several drugs in addition to antiplatelet agents have been reported to maintain VA patency. Treatment with dipyridamole plus low-dose aspirin substantially reduced the risk of stenosis and increased the duration of AVG patency [11]. However, one conflicting result of the effect of the same drug regimen on graft patency was reported [47]. Studies have revealed that statins reduced neointimal proliferation and vascular inflammation [9, 19, 26]. Until recently, direct evidence of a beneficial effect of statins on VA patency rate was not available [26]. In addition, low-dose warfarin does not appear to prolong AVG patency [27, 48]. Our findings revealed that statins, anticoagulants, and dipyridamole are ineffective at maintaining long-term AVF or AVG patency; these findings are consistent with those of some of the aforementioned studies. Nevertheless, a randomized controlled trial is required to firmly establish the role of these agents in VA outcomes.

### Limitations

Our study, similar to all database studies, had several inherent limitations. First, the data of several unmeasured confounders, such as drug compliance, use of self-paid medications, blood pressure, body mass index, cigarette smoking, physical activity, different types and combinations of antiplatelet agents, location of the VA (forearm or upper arm), and cannulation technique, are not available in the NHIRD. Second, previous studies have demonstrated the beneficial effects of some antihypertensive agents in prolonging primary VA patency, which were not included in our analysis [49]. However, we adjusted the comorbidity HTN in each group; consequently, balanced prescriptions of antihypertensive agents were anticipated. Third, laboratory data of inflammatory marker levels, lipid profiles, hemoglobin levels, and calcium–phosphate product levels were unavailable. Failure to consider the aforementioned variables may have caused some residual bias. However, because of the significance and magnitude of the observed effects, these limitations are unlikely to have noticeably affected the results. In addition, because the study cohort included only Taiwanese patients who received HD, the results may not be generalizable to other populations. Finally, our investigation was a retrospective observational study that had some methodological drawbacks and selection bias despite the case–control study design. The clinical relevance of this study must be further verified through large-scale prospective trials.

### Conclusion

The most noteworthy clinical implications of this study are that some risk factors are associated with early VA failure, and the use of antiplatelet agents protects VA from patency loss in a dose–response manner. Thus, active identification and treatment of risk factors and early administration of antiplatelet drugs are necessary in patients receiving dialysis that have multiple clinical risk factors.

## Supporting information

S1 File TMU IRB approval letter. (PDF)

S2 File Data of the NHIRD drug codes. (XLSX)
