# Correction: Antiplatelet agents maintain arteriovenous fistula and graft function in patients receiving hemodialysis: A nationwide case-control study

**DOI:** 10.1371/journal.pone.0215546

**Published:** 2019-04-17

**Authors:** Yung-Ho Hsu, Yu-Chun Yen, Yi-Chun Lin, Li-Chin Sung

There are errors in the author affiliations. The correct affiliations are as follows: Yung-Ho Hsu^1,2^, Yu-Chun Yen^3^, Yi-Chun Lin^3^, Li-Chin Sung^4,5^

**1** Division of Nephrology, Department of Internal Medicine, Shuang Ho Hospital, Taipei Medical University, New Taipei City, Taiwan, **2** Division of Nephrology, Department of Internal Medicine, School of Medicine, College of Medicine, Taipei Medical University, Taipei, Taiwan, **3** Research center of Biostatistics, College of Management, Taipei Medical University, Taipei, Taiwan, **4** Division of Cardiology, Department of Internal Medicine, School of Medicine, College of Medicine, Taipei Medical University, Taipei, Taiwan, **5** Division of Cardiology, Department of Internal Medicine, Shuang Ho Hospital, Taipei Medical University, New Taipei City, Taiwan

## References

[1] Hsu Y-H, Yen Y-C, Lin Y-C, Sung L-C (2018) Antiplatelet agents maintain arteriovenous fistula and graft function in patients receiving hemodialysis: A nationwide case–control study. PLoS ONE 13(10): e0206011 10.1371/journal.pone.0206011 30335833 PMC6193726

