# Supplementary material for: Antiplatelet agents maintain arteriovenous fistula and graft function in patients receiving hemodialysis: A nationwide case–control study
Source: PLoS One. 2018 Oct 18;13(10):e0206011. doi: 10.1371/journal.pone.0206011 (PMC6193726; doi:10.1371/journal.pone.0206011)
Supplement: S1 File — (PDF) [file pone.0206011.s001.pdf]

臺北醫學大學暨附屬醫院聯合人體研究倫理委員會

TMU-Joint Institutional Review Board

研究計畫免予審查證明

開立日期：民國105年09月14日

計畫編號：N201609023

計畫名稱：台灣血液透析病人瘻管存活時間的相關風險因子

計畫主持人：宋立勤

共同主持人：許永和

研究機構：臺北醫學大學、衛生福利部雙和醫院

研究人員：林怡諄

※請主持人依資料提供單位相關規定使用資料。

上述計畫經本會確認得免予審查，將於**第105-09-4次**會期核備，特此證明。

主任委員：

臺北醫學大學暨附屬醫院
聯合人體研究倫理委員會
Taipei Medical University
Joint Institutional Review Board

本會組織與執行皆符合適用法規
The TMU-Joint Institutional Review Board performs its functions according to written operating procedures and complies with GCP and with the applicable regulatory requirements.

c859cb4f002851422d289cdee02c0cf6

Certificate of TMU-JIRB

TMU-JIRB No. ： N201609023

Protocol Title： The risk factors associated with the vascular access survival time in hemodialysis patients in Taiwan.

Principal Investigator： Li-Chin Sung

CO- Investigator：Yung-Ho Hsu

Study Member：Lin,Yi Chun

Study Site:Taipei Medical University、TMU-Shuang-Ho Hospital

The above study has been confirmed Exemption by TMU-Joint Institutional Review Board.

Chairman：

Kuan-Jen Bai

臺北醫學大學暨附屬醫院
聯合人體研究倫理委員會
Taipei Medical University
Joint Institutional Review Board

本會組織與執行皆符合適用法規
The TMU-Joint Institutional Review Board performs its functions according to written operating procedures and complies with GCP and with the applicable regulatory requirements.
